# The role of gender relations in uptake of mass drug administration for lymphatic filariasis in Alor District, Indonesia

**DOI:** 10.1186/s13071-018-2689-8

**Published:** 2018-03-12

**Authors:** Alison Krentel, Kaye Wellings

**Affiliations:** 1 Department of Pathogen Molecular Biology, London School of Hygiene and Tropical Medicine, Faculty of Infectious and Tropical Diseases, Keppel Street, London, WC1E 7HT UK; 2 Bruyère Research Institute, 85 Primrose Avenue, Room 308-B, Ottawa, ON K1R 6M1 Canada; 3 Department of Social and Environmental Health Research, London School of Hygiene and Tropical Medicine, Faculty of Public Health and Policy, Keppel Street, London, WC1E 7HT UK

**Keywords:** Gender, Lymphatic filariasis, Indonesia, Compliance, Drug coverage, Social mobilisation

## Abstract

**Background:**

The Global Programme to Eliminate Lymphatic Filariasis has set 2020 as a target to eliminate lymphatic filariasis (LF) as a public health problem through mass drug administration (MDA) to all eligible people living in endemic areas. To obtain a better understanding of compliance with LF treatment, a qualitative study using 43 in-depth interviews was carried out in Alor District, Indonesia to explore factors that motivate uptake of LF treatment, including the social and behavioural differences between compliant and non-compliant individuals. In this paper, we report on the findings specific to the role of family and gender relations and how they affect compliance.

**Results:**

The sample comprised 21 men and 22 women; 24 complied with treatment while 19 did not. Gender relations emerged as a key theme in access, uptake and compliance with MDA. The view that the husband, as head of household, had the power, control, and in some cases the responsibility to influence whether his wife took the medication was common among both men and women. Gender also affected priorities for health care provision in the household as well as overall decision making regarding health in the household. Four models of responsibility for health decision making emerged: (i) responsibility resting primarily with the husband; (ii) responsibility resting primarily with the wife; (iii) responsibility shared equally by both husband and wife; and (iv) responsibility autonomously assumed by each individual for his or her own self, regardless of the course of action of the other spouse.

**Conclusions:**

(i) Gender relations and social hierarchy influence compliance with LF treatment because they inherently affect decisions taken within the household regarding health; (ii) health care interventions need to take account of the complexity of gender roles; (iii) the fact that women’s power tends to be implicit and not overtly recognised in the household or the community has important implications for health care interventions; (iv) campaigns and other preventive interventions need to take account of the diversity of patterns of health care decision-making and responsibility in specific communities so that social mobilisation messages can be tailored appropriately.

## Background

Lymphatic filariasis (LF) is an important public health problem facing low- and middle-income countries across the world. More than 120 million people worldwide are directly affected by the disease, and over a billion people are at risk of infection [1, 2]. LF is caused by thread-like parasitic worms living in the lymphatic system that can manifest as elephantiasis of the leg, scrotum, arm or breast. The disease results in long-term disability. Acute attacks may prohibit work and normal activity; elephantiasis of the scrotum and vulva interrupts sexual function; and the social exclusion and stigma attached to the disease brings about further distress [3].

Of the three types of lymphatic filariasis: *Wuchereria bancrofti*, *Brugia malayi* and *Brugia timori*, *B. timori* is found only in eastern Indonesia and so has been the focus of less research. In 1998, a Global Alliance for the Elimination of LF (GAELF) was created with the aim of eliminating the disease by 2020 through mass drug administration (MDA) [1]. A combination of a single dose of diethylcarbamazine citrate (DEC) or ivermectin (in those areas where onchoceriasis or loiasis is endemic) in combination with albendazole taken orally once a year for at least 5 years prevents progression of the disease in those already infected and prevents infection in those who are not [1, 4, 5]. To achieve elimination of lymphatic filariasis in Indonesia, mass treatment using DEC and albendazole must be given to at least 65% of the total population living in endemic areas, regardless of infection status, for at least 5 years [1, 6]. Coverage rates lower than this are not expected to eliminate the disease. Community awareness and education efforts are thus an essential part of the elimination programme. Convincing people to comply with a treatment which may have adverse reactions (in those infected with the microfilaria and adult worms) requires considerable efforts to mobilise communities and individuals, particularly in those areas where disease prevalence is low and people may have never seen the disease.

Social mobilisation has the potential to reduce fears and improve MDA participation but, to be effective, requires a clear understanding of what factors might enhance and hinder the inclination to take part in a MDA. Much of the research evidence thus far is drawn from political and geographical contexts outside of Indonesia. Reviews of factors associated with compliance have described issues related to both the individual and programmatic implementation [7–9]. Influences related to the individual include fear of adverse events [10], being male or female [10, 11], education level [12], and income [11]. Programmatic influences included degree of exposure to media campaigns and visits by local drug distributors [10, 13], in addition to being present on the day of drug distribution [10].

Through the published literature, there is evidence of differences in distribution and drug consumption between men and women. Women in Kerala State, India were more likely to take the LF pills than men were in a study by Regu et al. [14]. In other Asian-based research, women were more likely than men to receive the LF pills in the Philippines [15]. In two Indonesian districts, however, Krentel et al. [16] found that women were more likely to receive the pills during distribution, but were less likely than men to consume them [16]. This research is supported by similar findings in Uganda where men were more likely to miss the distribution than women due to being away from the house during the day when the drugs were delivered [17]. In Puri district in Odisha State, India, Hussain et al. [18] reported lower proportions in women than men for both receiving and consuming the LF drugs. These authors proposed that this difference may be due to higher literacy in males as well as school based distribution [18]. In research in Haiti, women were less likely to take the LF pills when compared to men, due to fears about possible interactions between the pills and their fertility [19].

Besides describing the differences in drug receipt and uptake between men and women, there has been little investigation into the impact of gender dynamics on participation and compliance in a LF MDA program, despite evidence that this may influence access to health care [20, 21] and preventive action [22]. Women’s access to resources and their bargaining power within the household have been shown to have a significant influence on their treatment seeking behaviour [23]. This paper explores the role of gender in the uptake of treatment for lymphatic filariasis through qualitative research.

## Methods

Alor is a small district with 13 islands in East Nusa Tenggara province, Indonesia. There are two types of LF in the district: *W. bancrofti* and *B. timori.* A survey in a highland area of Alor in 2002 showed a standardised population prevalence of 25% of microfilaria (mf) carriers for *B. timori* [24]. Due to vector breeding behaviours, *B. timori* filariasis is more prevalent in rice-growing areas and *W. bancrofti* filariasis is more prevalent in coastal areas [24].

In December 2001, the Indonesian Department of Health joined GAELF through the administration of a single dose of DEC/albendazole in endemic areas. Alor began MDA throughout the district in 2002. Compliance for MDA was reported to be 86.5% in 2002 and 90.6% in 2006 while the mf rate dropped from 25 pre-MDA to 0.3 in 2006 (Dinas Kesehatan District of Alor). Presently Alor District is in a post-surveillance phase monitoring for resurgence after stopping MDA in 2007 and completing the Transmission Assessment Survey (TAS) phase [25]. To reach and fulfil the requirements of the transmission assessment, Alor demonstrated its implementation of successful MDA rounds with high reported drug coverage (e.g. above the margin required for successful elimination). To obtain a better understanding of individual level compliance with LF treatment in this population, a qualitative study was carried out in Alor District. The aims of the study were to explore factors which appeared to motivate uptake of LF treatment, and to examine social and behavioural differences between compliant and non-compliant individuals. In this paper we report on the findings with specific reference to the role of family and gender relations. Gender roles and norms can be described as more traditional in Alor than in the more urban centres of Indonesia. A combination of Islam, Christianity and animism gives rise to further cultural differentiation within the island.

In total, 43 face-to-face in-depth interviews were carried out with a purposive sample of residents in both highly endemic areas for LF and in areas in which there was no reported LF. Participants were selected to achieve an equal balance of men and women, compliers and non-compliers and to represent both rural and urban areas. Determination of compliance was based on the last round of MDA. Systematic non-compliance was not a criterion for the interviews, so respondents could have switched from not taking, to taking the pills in the last round of MDA, qualifying them as compliers for the purposes of this research.

In-depth methods of investigation were designed to explore islanders’ reasons for accepting or rejecting the treatment offered. An unscheduled, unstructured topic guide was used throughout the interview. Topics addressed in the topic guide included causes and transmission routes of LF; knowledge and awareness of LF treatment; awareness of the local drug delivery system; influences on the decision to take or not to take the treatment; perceptions of the drug taking behaviours of others in the community; and perceived costs and benefits of the treatment and; perceptions about who is most likely to benefit from LF treatment.

**Fig. 1:**
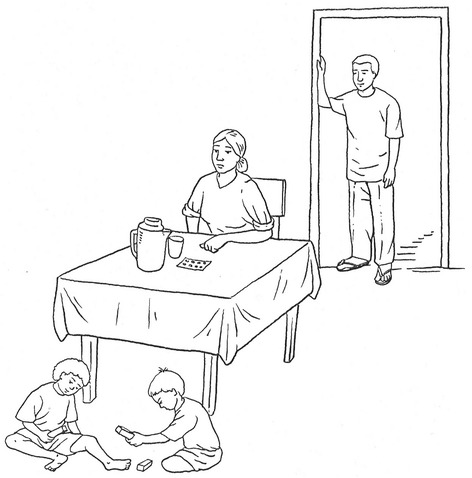
Drawing illustrating refusal or reluctance to take the LF treatment

**Fig. 2:**
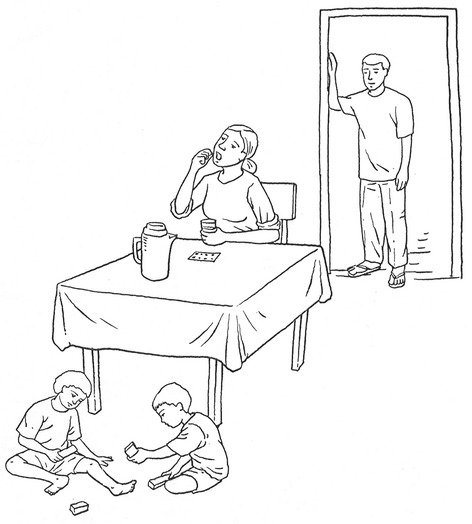
Drawing illustrating acceptance to take the LF treatment

In addition to the oral questions in the topic guide, participants were shown a set of illustrations depicting different scenarios relating to compliance or refusal (Figs. 1 and 2) and asked to describe what they thought was happening in the pictures. The illustrations depicted different drug taking scenarios within the household. One illustration depicted a woman seated at a table with a jug of water and a full glass in her hand in the process of taking the pills. Two children played in front of her and a man watched from the doorway. In a second illustration in the same setting, the woman sat with her hands on the table, apparently making no attempt to take the pills before her. Participants were asked to describe in their own words what was happening in each picture and to comment on the action. Towards the end of the interview, respondents were asked to rank a series of five statements in terms of importance. The statements were as follows: “*Take your pills so you don’t get filariasis*”, “*Take your pills so your children won’t get filariasis*”, “*Take your pills so our community doesn’t get filariasis*”, “*Take your pills so Alor doesn’t get filariasis*” and “*Take your pills so Indonesia doesn’t get filariasis*”.

To select respondents, the research team entered the village and went first to the health centre to inquire when the MDA had taken place and to locate neighbourhoods where possible compliers and non-compliers might be recruited. Based on that information, the team then chose one of the neighbourhoods in which the MDA had already taken place to begin the interviews. Where possible, the head of the village was informed of our research. The interviewer would enter the house, asking if someone in the household would consent to be interviewed. After general introductory questions, towards the beginning of the interview, the participant was asked about their participation (e.g. self-reported compliance) in the last MDA in their village. Interviews were conducted in a place of the participants’ choice. This was generally in their homes, but other venues included a shop and outdoors.

Interviews of approximately 1 h in duration were carried out by a western interviewer (AK) who had considerable working experience in Alor and spoke fluent Bahasa Indonesia. For a selection of interviews, an Indonesian social scientist assisted and provided additional assistance with translation, clarification and transcriptions. Interviews were taped with participants’ permission.

Data were analysed using ‘Framework’, a content analysis method of proven validity and reliability, which uses a thematic approach to classify and interpret qualitative research data [26]. Data from each interview were summarised in spreadsheet format. Two researchers (AK and KW) coded initial transcripts and discussed any disagreements in order to improve the reliability of coding.

## Results

### General characteristics of the sample

The sample comprised 21 men and 22 women; 24 complied with treatment while 19 did not. Three persons did not agree to their interview being recorded and these interviews were hampered by difficulty in language and understanding as these participants were fluent primarily in local languages. The findings presented in this paper are therefore based principally on 40 recorded interviews. The characteristics of the total sample are summarised in Tables 1 and 2.

**Table 1:** Characteristics of the sample: village location, environment, type of LF

Name of village (coded)	Distance from district capital (in hours by private car)	Environment	Kind of LF reported to be present	Total number of interviews	Men/ Women in sample
Village A	3	Mountainous, vanilla growing region	None	1	1 woman
Village B	< 0.5	Coastal	None	3	1 man/ 2 women
Village C	< 0.5	Coastal	None	3	2 men/ 1 woman
Village D	< 0.5	Flat land, rice growing	*B. timori*	6	2 men/ 4 women
Village E	0	Coastal	None	9	5 men/ 4 women
Village F	6	Coastal	*W. bancrofti*	7	3 men/ 4 women
Village G	4	Coastal and mountainous	*B. timori*	1	1 man
Village H	8	Coastal	*B. timori*, *W. bancrofti*	3	1 man/ 2 women
Village I	< 0.5	Coastal	None	7	3 men/ 4 women
Village J	1 ½	Mountainous, rice growing	*B. timori*	2	2 men
Village K	1 ½	Mountainous, rice growing	*B. timori*	1	1 man

**Table 2:** Characteristics of the sample: compliance and prevalence

	Self-reported compliance with last MDA treatment	Self-reported noncompliance with last MDA treatment
High prevalence areas	6 females; 5 males (11 total)	4 females; 5 males (9 total)
Low prevalence areas	5 females; 7 males (12 total)	7 females; 4 males (11 total)

Gender relations emerged as a key theme in access, uptake and compliance with mass drug administration (MDA). The view that the husband, as head of household, had the power, control, and in some cases the responsibility to influence whether his wife took the medication was common among both men and women. Gender also affected priorities for health care provision in the household as well as overall decision making with regard to health in the household.

In the following text, NC refers to non-compliant with the last LF treatment offered and C refers to compliant with the last LF treatment offered.

### Gender and responsibility for health

The evidence from the data suggests that Alorese men continue to be perceived as chief providers and as heads of the household, and this has implications for their perceptions of the importance of maintaining good health. A 48-year-old man (C) with 4 children living in village E explained that if he was sick and did not take any medication, then he would feel burdened because he had a wife and children. He added that he must be healthy in order to provide life’s necessities for his family. He explained, “*I am a father, if I am sick, everybody’s sick.*” As the sole provider for his family, any illness he may have, could seriously affect his family.

Another man (C), 43-year-old with 3 children living in village F reported that he himself had to be healthy before he could take care of others. When asked to prioritise people in terms of the importance of taking the treatment, he put himself first, then his wife; ensuring his own health first enabled him to guarantee the health of his household and his community. These men recognised their roles as chief provider and manager of their households, expressing the burden of this responsibility when they are unable to fulfil it.

There was also a strong sense of concern amongst men for their responsibility for the continuing survival of their family line. A 41-year-old farmer (C) from village D suggested that the best way to motivate people in his highly endemic village was to tell the men that they should ensure their lineage is healthy by complying with the treatment. He reported that it is the man’s responsibility for his lineage and future generations.

### Priorities for health care provision in the household

As the heads of the household in Alor, men are accorded priority in terms of receipt of health care and their needs were put before those of their wives and children. Interestingly, it was primarily women who held that men have the highest priority for health. Only one man, a farmer with 7 children (C), saw both his wife and himself as having the highest priority for health in their household. Many of these insights were revealed during the exercise in which respondents were required to arrange statements relating to prevention and cure of LF in terms of community, self, children, district and country in order of priority. The range of responses is represented below:

- Woman in her 20’s from village B, one child (NC): “My husband’s health is first, then myself, then my children’s.”
- 38-year-old woman with 6 children from village I (C): “I choose Indonesia as the first priority; Alor as the second; my husband, other family members and community as the third; my children as fourth; and myself as last.”
- 41-year-old woman with 5 children from village I (NC): “In the household, the father is the highest because he is the head of the household and responsible for arranging the wife and the children in the household. I consider as most important my husband because he is the head of the household, like I consider Jesus as the most important in my life and my husband is number 2. I have the opinion that I have to look after my husband’s health first.”
- 28-year-old woman with 2 children from village F (C): “Husband is in the number one priority place together with the wife, and then their children come afterwards.”

### Decision-making in relation to health

Four models of responsibility for health decision making emerged from the interviews: (i) responsibility as resting primarily with the husband; (ii) responsibility as resting primarily with the wife; (iii) responsibility as shared equally by both husband and wife; and (iv) responsibility as autonomously assumed by each individual for his or her own self, regardless of the course of action of the other spouse.

Regarding the first of these models, only one man in the sample, a father (NC) from village F, reported that in the household the father takes the health decisions. He added that if the father was not there, then the mother would take any decisions in his absence. He presented the husband as having prime responsibility for taking health-related decisions, suggesting a plan for when the man is not present in the home.

The second model, featured responsibility for health decision making as held by the women in the household. This model was reported more frequently in participants’ accounts than might have been expected, given the predominance of the husband as head of household and main decision maker. Yet the fact that women had the main responsibility for health appeared to be in keeping with the cultural context of Alor, where the women’s duty is to look after the household and its needs. This was illustrated in the account of a 48-year-old mother of 6 children (C) in village D:

*I: In your household, can you tell me who is responsible or concerned about health?*

*R: Health is the risk for us women to be responsible for. Looking after clothes*, *the interior of the house, and also the back of the house* [where the kitchen is located] *is the responsibility of the woman.*

*I: What about the man?*

*R: The man only knows how to call people to come over.* [Laughs] *Whatever there is, or isn’t, it is the responsibility of the housewife.*

*I: And if the children are sick?*

*R: Sick children, also, it is I who takes care of them.*

In this account, the woman actually jokes that her own husband is able to do little to assist her in the day-to-day running of the household. Her dialogue showed the difference between formal control (her husband calling people to the house) and informal control (taking care of the kitchen, sick children, laundry, etc.).

Two other women and one man held that it is the women’s responsibility to take care of health in the family. Their responses are presented below:

- Woman (NC), 53-year-old, 6 children, village E: “*The wife is involved and responsible for health in the family.”*
- Woman (NC), 41-year-old, 5 children, village I: “*In the household, it is usually the mother who has the responsibility to give the drugs to the husband and the children after they have eaten.”*
- Man (C), 41-year-old, 4 children, village E: “*He is not the boss of health in his household. It is his wife who takes more responsibility for health and reminding them of health.”*

These participants described ways in which women assume responsibility for health matters in their households: taking care of sick children, thinking about health, preparing food and drink, reminding their family members about health, taking decisions for health, instructing their children and giving medication.

The third model, that in which responsibility for decision-making regarding health is shared between men and women, was described in the accounts of six participants. Their shared responsibility for health reflects a pattern of greater gender equality. They tended to view health as too important a matter to be left to only one individual:

- Woman (NC), in her early 20’s, 1 child, village B: “Responsibility for health to me is all of ours and even more so in the household. We shouldn’t wait for the husband to say we need to take care of health.”
- Man (NC), 23-year-old, farmer, 1 child, village I: “In the household, the husband and wife together are responsible for health.”
- Woman (NC), 51-year-old, 7 children, village I: “Both husband and wife care about health - it is important.”
- Man (C), 43-year-old, 3 children, village F: “Health decisions are sometimes made by me, sometimes by my wife and sometimes together.”

Participants whose accounts fitted the fourth model of decision-making for health, in which individuals made autonomous decisions regarding treatment, were all from highly endemic villages for LF infection. A 25-year-old man (NC) from village D told me that his wife had complied with LF treatment and that he had neither forced her to comply nor prohibited her from doing so. Based on her experiences with side effects though, he decided against complying himself. The husband (C) of a 47-year-old woman also from village D had told his wife that she held her own fate and it was her own risk whether or not she wanted to take the treatment.

Where autonomous choices were made by man and wife, there was no guarantee that they would concur in their decision making and among some couples, there appeared to be some discordance between partners. A 28-year-old woman (C) in village F, took the treatment and encouraged her husband to do the same. When he laughed at her attempts to encourage his compliance, she told him the health staff would not give him drugs in the future if he needed them. A woman (C) with 7 children in village E claimed that if the wife wanted to take it and the husband does not, then each one was on his/her own. A 37-year-old farmer (C) from village H remarked how his wife was an adult and could take it on her own. Among these apparently discordant couples, the predominant pattern was for the wife to take the drug while the men declined. Some of the men in this category however, despite being strongly against compliance, did not interfere with their wives’ decisions to take the drugs.

### Gender relations, power and influence

Participants had a further opportunity to describe the power balance between husband and wife, and its influence on compliance, within a fictional household. As noted in the methods section, participants were asked to describe drawings in which a woman was seated at a table with a packet of medication and a glass of water in front of her. In discussions of the hypothetical situations in the drawings, participants made references to the use of force, or the threat of force, by men. Both men and women used words such as “order”, “have to take it”, “must” or “is not allowed” - usually to describe the man’s command to the woman. These words reveal imperatives rather than persuasion. A man (C) from highly endemic village J said that the woman in the drawing *must* drink the treatment because it is there to overcome the disease, adding that the man had already drunk the medication so his wife *must* too, to prevent the disease being transmitted to the family. When explaining what was happening in the pictures, several men described the fictional husband’s right and responsibility, as head of the household, to give orders to his wife to comply or not to comply with the MDA and the husband’s greater knowledge, while women told of the wife’s inability to refuse her husband’s wishes. In describing the pictures, the respondents painted a picture of male authority. Comments on the hypothetical situation revealed perceptions of a heavier handed approach on the part of men compared with the accounts people gave from their own experiences (one man (C) even interpreted the husband’s intentions being to hit his wife). These portrayals of male authority seem to contradict participants’ accounts relating to decision-making about health within their own households. Comments from four individuals illustrate these contradictions. Each, in their real-life accounts, spoke about joint responsibility for health. Yet each spoke of force when describing the fictional situation in the drawings. One, a woman (NC) from village B, commented in relation to her real-life situation “*we should not wait for the husband to say we need to take care of health*”, whilst acknowledging the man’s authority in the hypothetical example stating that the woman cannot refuse her husband’s force. Similarly, a farmer (NC) from village I expressed his own commitment to joint responsibility but spoke of the fictional husband being able to order his wife to comply because of his responsibility for the household. A university educated man (C) from village E reported that he was not the boss of health in his household rather that his wife was. He interpreted the drawings, however, in terms of the woman being forced by her husband to comply. He acknowledged that his description of the drawings differed from his description of his own experience. When asked what would happen if the fictional woman refused, this participant hesitated as he had rarely seen evidence of that and so could not answer.

How can these apparent contradictions be interpreted? It appears that some women in Alor do not enjoy the same degree of freedom as their husbands. Curtailment of women’s movements by husbands does occur. For example, a 30-year-old (NC) woman from village E reported that her husband did not allow her to go to her neighbours’ houses in case she might gossip with them. Such restrictions may not directly affect compliance with the MDA, but they would prohibit potential conversations with neighbours about the treatment that may be beneficial in terms of creating social norms favouring compliance. The protected status of women and their confinement to the household and to family duties was also, in some cases, a barrier to access to the drugs. One man (NC) told me that in his house, no one took the treatment because he did not go to get the drugs. His family had no opportunity to comply because there were no pills. A woman (NC) from village B also told of her husband’s ‘gatekeeping’ actions: when picking up the drugs for the family, he told the health staff his wife was breastfeeding and as a result, they did not give him drugs for her. She accepted his decision without question. At the same time, it seemed beyond men’s power or persuasion to control their wives’ every movement. A 38-year-old with 3 children (NC), was unsure whether his wife took the treatment, but surmised that she probably had not done so because he was suspicious of her taking the medication in her current pregnant condition. He added that he would not have forced her to comply anyway; he had seen information on women’s empowerment on television and that he would not want to be reported for forcing her. He laughingly added that he did not understand enough about it.

## Discussion

These accounts reveal the complexity of gender relations in Alorese society. Alorese men are perceived to be, and perceive themselves to be, the head of the household and the chief provider, and as a result are prioritised in terms of health care. The role of Alorese women, however, is to take care of the household needs, including those of the husband and children. In many instances responsibility for health care decision making is taken solely by women, and sometimes jointly with the husband. Further, whilst respondents’ descriptions of hypothetical situations relating to compliance with treatment appear to authorise the use of coercion by men, this notion appears to be less frequently reported in the context of everyday experiences. At the same time, real-life accounts reveal that control is still exercised by some men over the access their wives have to LF treatment. There is also evidence of considerable diversity in patterns of gender relations within Alorese households. With regards to responsibility for health in the household, the patterns described fitted four different models in relation to health care decision making, that is, responsibility being vested in men; in women; in men and women jointly; and by each for him or herself. The complexity and the diversity in patterns of gender roles reflect the changing nature of Alorese society. While much of Alorese culture remains rooted in the more traditional, particularly in the rural areas, there are nonetheless signs of transition from a traditional, patriarchal society to a more modern egalitarian society. It is possible that the tendency to describe real life situation as less patriarchal, and the fictional scenario as more so, may reflect this gradual transition from the older to the newer form of social organisation. But it seems also to be the case - as we have seen in the co-existence of four quite different models of gender patterning in decision making - that elements of the two orders, old and new, exist side by side in contemporary Alorese society. Alorese people are still able to relate to the old system and to describe its norms regarding traditional gender relations; but they are increasingly exposed to a more modern culture and to begin to modify their own household and thinking according to this newer norm.

This research is not without limitations. Since opportunities to observe directly the process of taking the tablets are few, this study relies on self-reported accounts of compliant behaviour, making the study susceptible to biases relating to recall and veracity of response. The research was conducted at the same time the MDA was ongoing in the district, so to reduce recall bias, one of the criterions for the village selection was that they had recently received the last round of MDA for that year. Despite efforts to limit recall bias, some people still may have incorrectly recalled their compliance and confused it with campaigns in previous years. The gender of the primary interviewer (AK) may have influenced the validity of the data collected. It is possible that in some interviews with men, a more public account was received [27] versus the interviews with women where there was greater ease and openness in the discussion. Nonetheless, our data find resonance in the reports of other researchers. In some respects, the picture with regard to gender relations in Alor seems to have changed little since Cora DuBois described it in 1944 [28, 29]. DuBois suggested that the recognition of women’s contributions to the household were implicit rather than explicit [29]. Our data also resonate with the more recent observations of Utomo [30] relating to gender relations in Indonesia, that “women’s *noble role”* (page 2) in Indonesia relates to their function as wives and mothers as their first priority. Utomo’s descriptions of patterns among the urban middle-classes in Jakarta, however, show changes in social norms regarding women’s role in the household, demonstrating a preferential shift towards a more equal marriage [30]. This is consistent with the evidence from our data of joint responsibility and autonomy in decision-making among some respondents in Alor and may be attributed to the ongoing development of the district and the increased influence of modernisation and consumerism. These trends are likely to continue, particularly with the increasing availability of education across Indonesia for women. Beegle et al. [31] describe education as way for women to argue for the adoption of modern behaviours in everyday life. Separate ownership of assets and education, the social status of the woman and the education levels of the fathers (woman’s father and her father-in-law) all contribute to the woman’s ability to make decisions in regards to her reproductive health [31]. These factors may also offer an explanation as to why different models of responsibility for health care decision-making co-exist in our sample. The status of women and their influence on health in the household has also been documented in relation to infant and child mortality in West Java [32].

### Implications of the findings for LF elimination

Our data provide insights into the nature of gender relations and their implications for compliance with MDA for LF elimination. Although these relate specifically to the Alorese context, they are likely to have value for LF programmes in other areas, in particular those in rural patrilineal societies. First, at the most general level, it can be seen that gender relations and the social hierarchy influence compliance with LF treatment because they inherently affect decisions taken within the household regarding health. Intervention efforts to encourage compliance need to heed the fact that the natural inclination in Alorese society might be to give priority to protecting men’s health and so extra efforts may be needed to stress the need for women to remain healthy too and to see their own health as a priority. In circumstances where women’s access to treatment may be limited (e.g. the husband does not pick up the drugs), women can be encouraged to act for their own health and the health of their children, as a way of maintaining a healthy family and its future. This concept could also be integrated with men’s desire to ensure a healthy lineage. Men can be encouraged to see the health of their wives and children as a priority to accomplish their own goals as head of the household in terms of continuation of lineage and as a result should make every effort to ensure that their household receives treatment. Secondly, health care interventions need to take account of the complexity of gender roles. Men are traditionally the heads of household and providers, but women are essentially responsible for the day to day life in the family, taking responsibility for the care of children, much of the planting and harvesting, cooking and cleaning and also, as many of these accounts suggest, for health care in the family. While the man retains the public image as head of household and may control the process of collecting the medication, it is likely the wife who will administer the medication to the family ensuring that they all (including the husband) take it correctly. It is important to accommodate the nuances in this division of roles in campaigns designed to increase compliance in a community. Approaching men ensures that there is the will or desire to comply with treatment within the household, whereas approaching women ensures that the drugs will be administered in the household. Both approaches are needed for successful uptake.

Thirdly, the fact that women’s power tends to be implicit and not overtly recognised in the household or the community has important implications for health care interventions. Programmes need to preserve men’s sense of power and avoid making them feel that their power and authority is being usurped, while at the same time acknowledging that women have de facto power at the household level when it comes to responsibility for health issues. Finally, campaigns and other preventive interventions need to take account of the diversity of patterns of health care decision-making and responsibility in specific communities. The fact that quite different models in terms of gender roles may exist side by side even in a relatively small community means that messages and approaches must be targeted and tailored to multiple audiences with specific communicational and motivational needs.

## Conclusions

These analyses have shown the importance of collecting data on the social context, including gender dynamics, in which health care interventions are implemented. Comprehensive data on values and attitudes are essential to the careful targeting of interventions and to the tailoring of messages to specific population sub-groups, in terms of their tone and content. Our research also reminds us of the importance of devising methodologies which provide insights, not only into practices amongst individuals and within households, but also into the broader social norms governing action, which may at times be discrepant. The findings of such studies provide a solid evidence base for policy making in key areas of public health and prevention. Finally within the context of LF elimination, increased understanding of gender relations in the household and in the community is required to facilitate successful mass drug administration.

## Abbreviations

C: Complier
DEC: Diethylcarbamazine citrate
LF: Lymphatic filariasis
MDA: Mass drug administration
NC: Non-complier
